# Simultaneous ThermoBrachytherapy: Electromagnetic Simulation Methods for Fast and Accurate Adaptive Treatment Planning

**DOI:** 10.3390/s22041328

**Published:** 2022-02-09

**Authors:** Ioannis Androulakis, Rob M. C. Mestrom, Miranda E. M. C. Christianen, Inger-Karine K. Kolkman-Deurloo, Gerard C. van Rhoon

**Affiliations:** 1 Department of Radiotherapy, Erasmus MC Cancer Institute, University Medical Center Rotterdam, 3015 GD Rotterdam, The Netherlands; m.christianen@erasmusmc.nl (M.E.M.C.C.); i.kolkman-deurloo@erasmusmc.nl (I.-K.K.K.-D.); 2 Department of Electrical Engineering, Eindhoven University of Technology, 5600 MB Eindhoven, The Netherlands; R.M.C.Mestrom@tue.nl; 3 Department of Radiation Science and Technology, Delft University of Technology, 2629 JB Delft, The Netherlands

**Keywords:** interstitial hyperthermia, automated treatment planning, electromagnetic simulations, ThermoBrachytherapy, high dose rate brachytherapy, quasistatic simulations, capacitive heating, treatment plan optimization, finite-difference time-domain, gamma index analysis

## Abstract

The combination of interstitial hyperthermia treatment (IHT) with high dose rate brachytherapy (HDR-BT) can improve clinical outcomes since it highly enhances the efficiency of cell kill, especially when applied simultaneously. Therefore, we have developed the ThermoBrachy applicators. To effectively apply optimal targeted IHT, treatment planning is considered essential. However, treatment planning in IHT is rarely applied as it is regarded as difficult to accurately calculate the deposited energy in the tissue in a short enough time for clinical practice. In this study, we investigated various time-efficient methods for fast computation of the electromagnetic (EM) energy deposition resulting from the ThermoBrachy applicators. Initially, we investigated the use of an electro-quasistatic solver. Next, we extended our investigation to the application of geometric simplifications. Furthermore, we investigated the validity of the superpositioning principle, which can enable adaptive treatment plan optimization without the need for continuous recomputation of the EM field. Finally, we evaluated the accuracy of the methods by comparing them to the golden standard Finite-Difference Time-Domain calculation method using gamma-index analysis. The simplifications considerably reduced the computation time needed, improving from >12 h to a few seconds. All investigated methods showed excellent agreement with the golden standard by showing a >99% passing rate with 1%/0.5 mm Dose Difference and Distance-to-Agreement criteria. These results allow the proposed electromagnetic simulation method to be used for fast and accurate adaptive treatment planning.

## 1. Introduction

In the treatment of prostate cancer, interstitial high dose rate brachytherapy (HDR-BT) is commonly used either as monotherapy (mainly for low and favorable intermediate-risk prostate cancer) or in combination with external beam radiotherapy (in high-risk prostate cancer) [1]. Recently there have been attempts to further hypofractionate HDR-BT monotherapy for prostate cancer, with positive results down to a two-fraction treatment protocol [2]. Attempts to deliver an adequate radiation dose to the target in these ultra-hypofractionated treatments have increased stress on the neighboring organs at risk (OAR), and so far, attempts to go to a single fraction monotherapy treatment have shown discouraging results [3,4]. While generally, the focus is on increasing the physical radiation dose, an interesting alternative is to aim at increasing the biologically effective dose (BED), with the focus on sensitizing the target more than the surrounding tissue and OAR.

One of the most potent sensitizers to radiation is hyperthermia (HT) [5]. Specifically for the prostate, the benefit of combining radiation with hyperthermia is shown in the clinic in a recent retrospective comparative study [6]. When HT is used before or after radiotherapy (RT), it sensitizes tumor cells to RT, while the healthy cells quickly restore to their original sensitivity to RT [7,8]. When HT is used simultaneously with RT, thermal sensitization is much higher, both for tumor and normal tissue [9]. Whether simultaneous RT+HT is superior over sequential RT+HT depends on the ability to preferentially deliver the RT and HT to the target, aiming at achieving maximum protection of the healthy surrounding tissue and OAR. Very local heating can be performed with interstitial hyperthermia (IHT) devices. The benefit of using interstitial hyperthermia together with interstitial brachytherapy has been shown in vivo [10], while clinical studies have shown good heating characteristics in sequential HDR-BT and IHT prostate cancer treatment [11].

To introduce such highly enhanced simultaneous RT+HT treatment, we recently developed ThermoBrachy applicators. The ThermoBrachy applicators provide the required hardware for simultaneous delivery of highly localized HDR-BT and electromagnetic (EM) interstitial hyperthermia [12]. Hence, these applicators have the potential to seamlessly integrate IHT in the HDR-BT treatment process, pursuing the high enhancement of the radiation dose in the target volume. However, a fast and accurate HT treatment planning platform that can perform IHT treatment planning in similar times as for HDR-BT is mandatory for effective integration. In achieving this goal, the most considerable challenge is to achieve a drastic simulation time reduction (from several hours to seconds) for the EM power deposition. A critical obstacle in this process is that the ThermoBrachy applicators have a small diameter (2 mm) with extremely thin layers (several μm) of conductive and dielectric material. The μm-sized features make accurate modeling computationally expensive. This is especially true in real patient scenarios where the applicators are usually not perfectly parallel to each other, and hence, more voxels are necessary to accurately discretize the applicator structures [13]. In addition, the most commonly used calculation method—Finite Difference Time Domain (FDTD)—is an inefficient technique at the low operating frequency of the ThermoBrachy applicators (27 MHz). Finally, for treatment planning, it is essential to compute the field distributions separately for each electrode, as these are subsequently used as inputs to an optimization algorithm [14].

Hyperthermia treatment planning for IHT is generally uncommon, mainly due to the complexity induced by the small structures of the applicator [13]. In an earlier developed capacitively coupled IHT applicator, the Multi-Electrode Current Source (MECS) applicator, a homebuilt quasistatic energy deposition model was used. This model defines the applicators independently from the grid, making it compatible with implants that are not parallel to the computational grid [15]. Further IHT Treatment Planning (TP) has been investigated mainly in interstitial ultrasound applicators [16]. Moreover, no commercial TP software in EM-based IHT is available for patient-specific treatment planning in IHT.

In this study, we report the development and validation of a fast and accurate computation method to predict the EM field produced by the ThermoBrachy applicators using commercial simulation software. We first validate that a quasistatic approximation can be used for the thermobrachy applicators. Secondly, we apply and investigate the accuracy of an applicator model approximation that severely reduces the model complexity and computation time. Thirdly, we evaluate the applicability of the concept of electric field superpositioning, which can be utilized by a field optimization algorithm. All steps are validated using γ-index analysis. Finally, we demonstrate that we can combine these methods to achieve fast and accurate treatment planning in realistic patient models.

## 2. Materials and Methods

### 2.1. Hyperthermia System

**Figure 1:**
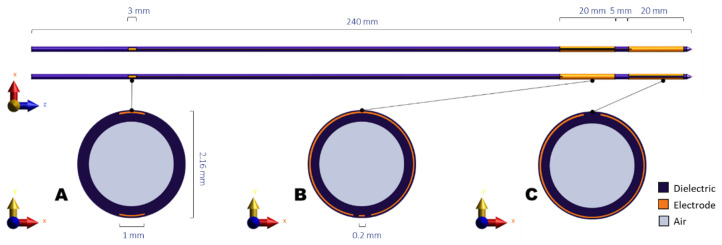
Schematic representation of the ThermoBrachy applicator. (**A**–**C**) show cross-sections of the applicator at the level of the connector patches, the proximal electrode center, and the distal electrode center, respectively.

The Thermobrachy applicator is a dual-electrode capacitively coupled IHT applicator where each electrode is 20 mm long, and the distance between the two electrodes is 5 mm. The electrodes are placed around a hollow polyoxymethylene (POM) catheter of 2 mm outer diameter and 1.66 mm inner diameter. Copper connection lines lead radiofrequency (RF) current from the posterior end of the catheter to the copper electrodes. The whole applicator and conductive layers are covered by a Parylene C dielectric layer to avoid galvanic contact between the conductive layers and the tissue. A graphic representation of the applicator design is shown in Figure 1. A proof of principle has been presented in our previous work [12].

While the ThermoBrachy applicators can be used at other treatment sites where interstitial HDR-BT can be performed, in this study, the case study site is the prostate. The ThermoBrachy applicators are directly inserted into the prostate identical to conventional 6F HDR-BT catheters according to the recommendations in [1]. An averagely sized prostate is typically implanted with 16 to 22 applicators. The applicators are meant to be simultaneously connected to the HDR-BT afterloader and the 27 MHz IHT RF-power generators, allowing for simultaneous irradiation and hyperthermia.

For the EM energy deposition that generates the temperature increase, all electrodes that are positioned inside the target are provided with an in-phase 27 MHz RF signal. The phase of the signal can change between 0° and 180°, while the power of the signal can vary from 0 to maximum power. The selected phase and power per electrode are defined based on an optimization process that requires the knowledge of the specific absorption rate (SAR) distribution that is produced by each separate electrode inserted into the patient.

### 2.2. SAR Calculation

Four methods were used in this study to calculate the three-dimensional electromagnetic solutions with the ThermoBrachy applicator, and hence calculate the three dimensional SAR distributions in phantom or patient models:

1. Single calculation with a full-wave Finite-Difference Time-Domain (FDTD) solver, applied on a detailed model of the electrodes in the phantom setup;
2. Single calculation with an electro-quasistatic (EQS) solver applied on a detailed model of the electrodes in the phantom setup;
3. Single calculation with an EQS solver applied on a simplified model of the electrodes in the phantom setup;
4. A superpositioning of all electric fields, calculated separately with the EQS solver for each electrode, using the simplified model of the electrodes in the phantom setup.

As a ground truth SAR distribution, we used the results given by the detailed full-wave FDTD solver (calculation method 1). This calculation method has been used extensively in HT TP and applies to the whole EM spectrum, and has no setup restrictions [17]. The FDTD solver has also been verified for the ThermoBrachy applicators in our previous work [12].

All simulations were performed using the Sim4Life, version 6.2, ZMT, Zurich, Switzerland. For the FDTD simulation, a CUDA GPU accelerated solver was used, utilizing three GeForce GTX 1080 Ti graphics cards. For the model generation, voxeling, and EQS simulations, no GPU acceleration was used, and the calculations were performed on an Intel Core i7-6700 CPU with 16GB of RAM. The approaches for the four different simulation methods will be described in the following subsections.

#### 2.2.1. Calculation Method 1: FDTD Solver Applied on the Detailed Model

We used the EM full-wave FDTD solver for the accurate calculation of the specific absorption rate (SAR) distributions [18]. A non-uniform grid was used in the simulations. To accurately discretize all layers of the applicators, a maximum grid step of 0.02 mm was chosen to discretize the cylindrical cross-section of the applicators (x, y). In the regions outside the applicators, the grid was gradually increased to a maximum of 2 mm). Along the longitudinal axis of the applicators (z), a grid step of 1 mm was used. An edge source with a frequency of 27 MHz, a voltage of 20 V, and a load of 50 Ω was used between the edges of each of the feeder wires and a ground. All copper wires and electrodes were simulated as perfect electric conductor (PEC) materials. The properties used for all other materials and tissues can be found in Table 1. A Uniaxial Perfectly Matched Layers (UPML) absorbing boundary condition was selected at the boundaries of the simulation domain. The minimum distance between the boundaries and the applicators was set to 10 mm to not interfere with the electric field generated around the applicators. The single FDTD simulation will be referred to as calculation method 1 in the rest of the document.

#### 2.2.2. Calculation Method 2: EQS Solver Applied on Detailed Model

The ThermoBrachy applicators in the IHT application operate at a frequency of 27 MHz. In tissue, this yields a wavelength of over a meter, which is two orders of magnitude larger than the size of the applicators. Therefore, the EQS approximation:

(1) $$\nabla \cdot \left(\sigma +j\omega \varepsilon \right)\nabla V=0,\;$$

can be used, where *σ* is the electrical conductivity, *ω* is the angular frequency, V is the scalar electric potential, and *ε* is the dielectric permittivity [19].

We used an EQS finite element method (FEM) solver for the calculation of the electric field and SAR distributions [20] at 27 MHz. The same non-uniform grid as described for the FDTD solver was used. A constant voltage (Dirichlet) boundary condition was applied on the active electrodes, while a constant 0 V boundary condition was imposed on the boundaries of the computational model, which were set at 10 mm distance from the volume of interest boundaries, in order not to interfere with the electric field generated around the electrodes. The properties used for all materials and tissues can be found in Table 1. The electrode voltage was adapted to 13 V to match the potential observed at the electrodes in the FDTD simulations. This single EQS simulation will be referred to as calculation method 2 in the rest of the document.

#### 2.2.3. Calculation Method 3: EQS Solver Applied on a Simplified Model

**Figure 2:**
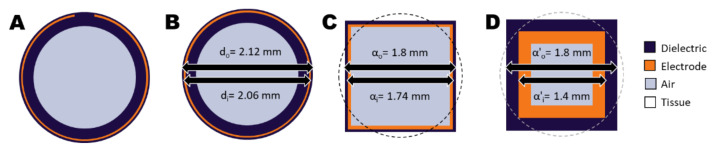
Cross-sections of the ThermoBrachy applicator show the geometric simplification steps applied in this study. (**A**) The actual cross-section of the thermobrachy electrode. (**B**) The electrode can be closely approximated by a cylindrical electrode equivalent. (**C**) The cylindrical applicator can be closely approximated by an orthogonal equivalent with equal outer surfaces for the electrode and dielectric layers. (**D**) The dimensions of the orthogonal layers can be adapted with a simultaneous adaptation of the dielectric properties of the materials. In all subfigures, tissue is represented by the background color (white) and surrounds the applicator.

The electrodes of the applicators form a capacitor with the tissue on the outer side of the dielectric material (see Figure 2A). A close approximation of the geometry is that of two concentric cylindrical shells, where the inner cylindrical shell with an outer diameter of 2.06 mm is the electrode and the outer cylindrical shell with an outer diameter of 2.12 mm is the dielectric material (Figure 2B). The impedance of two concentric cylindrical conductors can be derived as

(2) $$Z=\frac{ln\frac{d_{0}}{d_{i}}}{2\pi l\left(\sigma +ij\omega \varepsilon \right)}\;,$$

where $d_{0}$ and $d_{i}$ are the outer and inner diameters, respectively, and l is the length of the electrode.

In the situation of the ThermoBrachy applicator, the real part of the impedance is much lower than the tissue impedance, which makes the electrode encircled by the dielectric material act as a current source [25]. As the computational approach is voxel-based, a speed-up can be gained by simplifying the applicators to a cuboid equivalent with a similar volume (Figure 2C). For this, we chose a geometry with an equivalent length and electrode surface. The dielectric layer between tissue and electrode is very thin, resulting in a need for very detailed voxeling. The dependence of the capacitance on the dielectric thickness can be derived from Equation (1) and is equal to

(3) $$C=\frac{2\pi \varepsilon l}{ln\frac{d_{0}}{d_{i}}}\;,$$

for the coaxial geometry. Similarly, it is

(4) $$C=\frac{4\varepsilon l}{ln\frac{\alpha_{o}}{\alpha_{i}}}\;,$$

for the cuboid geometry of Figure 2C, where $\alpha_{o}$ and $\alpha_{i}$ are the outer and inner edges of the coaxial cuboid capacitor. In both cases, it is possible to obtain the same capacitance by changing the lateral dimensions of the electrodes ($\alpha_{ο}^{\prime }$, $\alpha_{i}^{\prime }$), if this is compensated by a proportionally adapted dielectric permittivity ($\varepsilon^{\prime }$) for the dielectric material. Therefore, a configuration like that in Figure 2D is expected to deliver the same electric field results outside the applicator if $\varepsilon^{\prime }$ gets the value:

(5) $$\varepsilon^{\prime }=\varepsilon \;\frac{\ln\frac{\alpha_{ο}^{\prime }}{\alpha_{i}^{\prime }}}{\ln\frac{\alpha_{o}}{\alpha_{i}}}$$

We applied the EQS FEM solver on a simplified rectangular applicator model with $\alpha_{i}^{\prime }=$ 1 mm and $\alpha_{ο}^{\prime }=$1.4 mm for the electrode, and $\alpha_{i}^{\prime }=$ 1.4 mm and $\alpha_{ο}^{\prime }=$1.8 mm for the dielectric layer. A non-uniform grid, as before, was used in the simulations. A coarser grid step of 0.1 mm was chosen to discretize the rectangular volume in the perpendicular plane of the applicators (x, y), while the rest of the grid settings remained, as stated before. The simplified single EQS simulation will be referred to as calculation method 3 in the rest of the document.

#### 2.2.4. Calculation Method 4: Superpositioning of EQS Solver Results Applied on a Simplified Model

The SAR can be calculated from the electric field (E) distribution using the following relationship:

(6) $$SAR=\frac{1}{2}\sigma {\left|E\right|}^{2}\;$$

For treatment planning optimization, the electric field distribution $E_{i}$ can be calculated for every electrode (*i*) separately. Then the total electric field distribution resulting from any combination of electrode amplitudes $n_{A}$ and polarities $n_{\varphi }$ can be derived by superpositioning of the separate electric field distributions as

(7) $$E_{tot}=\overset{\forall \;electrodes}{\underset{i}{\sum }}n_{A,i}n_{\varphi ,i}E_{i}\;$$

This means that the total electric field $E_{tot}$ resulting from any combination of $n_{A}$ and $n_{\varphi }$ can be calculated through superposition, if all $E_{i}$ are already pre-calculated. This is especially helpful if multiple electrode settings need to be evaluated.

Using the model and solver settings described in Section 2.2.3, we calculated the electric field distribution for each electrode by leaving the voltage of the respective electrode at its original potential of 13 V and all the other electrodes at zero potential. Then we combined all the electric fields to generate the total electric field according to Equation (7). The SAR resulting from the electric field was calculated using Equation (6). The simplified superpositioned model EQS simulation will be referred to as calculation method 4 in the rest of the document.

### 2.3. Validation of SAR Calculations

**Figure 3:**
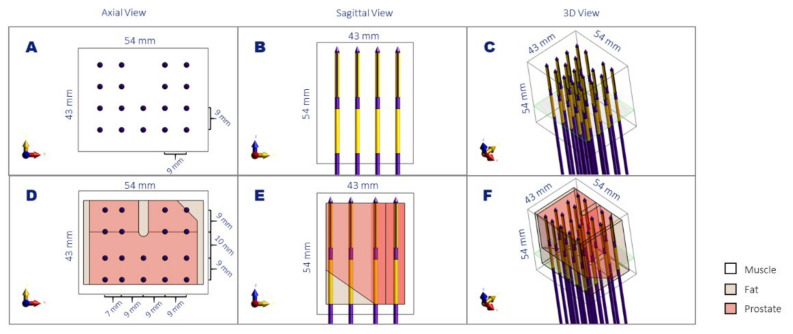
Schematic representation of the homogenous (**A**–**C**) and complex (**D**–**F**) benchmark models in axial view (**A**,**D**), sagittal view (**B**,**E**), and a 3D view (**C**,**F**). The noted dimensions correspond to the volume of interest used for the simulation comparison and the distance between the applicators. The plane denoted in green corresponds to the slice visualized in Figure 4.

To validate the four simulation methods, we generated two three-dimensional benchmarking models. In the first benchmark model, 18 applicators were placed parallel to each other, with a homogeneous 9 mm spacing in homogenous muscle tissue (Figure 3A–C). The rationale behind choosing 9 mm homogenous spacing was that in HDR-BT, it is recommended to implant the catheters at a maximum distance of 10 mm [1]. This homogeneous benchmark model has dimensions close to a prostate model and a shape that resembles the real prostate brachytherapy scenario (no electrodes in the upper central part, where the urethra is placed), but due to its symmetry, it allowed us to apply the two detailed model calculation methods (calculation methods 1 and 2) with realistic model sizes and reasonable computation times for all simulation methods. A second, more complex benchmark model was created (Figure 3D–F), which includes heterogeneity in tissue properties (both perpendicular and in parallel to the applicators) and has a less symmetric applicator configuration. The model has fat, muscle, and prostate tissue, with properties according to Table 1, in a way that closely resembles the situation that can be present in an actual patient. Namely, applicators passing through a horizontal prostate-fat interface; an applicator placed midway through a prostate-fat interface; and distance variations between the applicators. The applicator orientations were kept parallel to one of the orthogonal axes to accomplish realistic model sizes and computation times. Note that when the thin layers of the applicator are not aligned to the orthogonal axes, the number of voxels needed to discretize the applicator structure can increase considerably (see chapter 3). An equal input power of 0.7 W was applied on all electrodes in the benchmarking calculations. The former benchmarking model will be referred to as the Homogeneous Benchmarking model, while the latter will be referred to as the Complex Benchmarking model in the rest of the document.

As an evaluation metric, we used γ-index analysis [26]. This method has been extensively used in radiotherapy [27,28] and HT [29,30] for field comparisons, mainly between calculation and measurement, but also between two different calculation methods. We applied the 3D evaluation algorithm as applied by de Bruijne et al. [29]. The level of agreement between the evaluated calculation and reference calculation is expressed by the percentage of voxels that have a γ-index <1 for a chosen SAR dose difference (DD) and a chosen distance to agreement (DTA). For the computation of the γ-index, the reference field is compared point-wise to nearby points in the evaluated field to compute the generalized Γ value for each cell in the reference field. Γ is computed as

(8) $$\Gamma \left(\vec{r_{e}},\vec{r_{r}}\right)=\sqrt{\frac{{\left(SAR\left(\vec{r_{r}}\right)-SAR\left(\vec{r_{e}}\right)\right)}^{2}}{DD^{2}}+\underset{j=x,y,z}{\sum }\frac{{\left|\vec{r_{r}}-\vec{r_{e}}\right|}^{2}}{DTA^{2}}}\;,\;$$

where $\vec{r_{r}}$ and $\vec{r_{e}}$ are the reference and evaluated points in space, respectively. Then the γ value at point $\vec{r_{r}}$ is the minimum of the generalized Γ function:

(9) $$\gamma \left(\vec{r_{r}}\right)=\min\left\{\Gamma \left(\vec{r_{e}},\vec{r_{r}}\right)\right\}\forall \vec{r_{e}}$$

As a volume of interest (VOI) for our calculations, we used the 54 mm × 43 mm × 54 mm rectangular volume, including the active lengths of the applicators. In all calculations, we regarded calculation method 1 as a reference. According to the AAPM Task Group 186 report on model-based dose calculation methods in brachytherapy, the minimal requirement for a good agreement is a 99% passing rate for 2%/2 mm DD and DTA [31]. In addition to that, a 1%/0.5 mm DD and DTA were used. We also report voxel-wise spatial accuracy (mean absolute error) and bias (mean error) as a percentage of the maximum SAR in the VOI. Table 2 gives an overview of the calculation methods evaluated with the benchmarking models.

### 2.4. Application on Real Patient Scenarios

To demonstrate the feasibility of treatment planning in a real patient scenario, we applied our simplified IHT calculations on the anatomic and implant data of 3 patients treated with HDR-BT for prostate cancer. We used the brachytherapy planning CT image for the target (prostate) and OAR delineation as well as for needle reconstruction. The prostate, urethra, rectum, and bladder volumes were contoured from the segmentation used for HDR-BT treatment planning. For muscle, fat, and bone volume segmentation, an automatic segmentation workflow was used.

The simulated ThermoBrachy applicators were positioned in the exact location as the HDR-BT catheters. The tip of the HDR-BT catheters was identified in the CT images, and the tip of the ThermoBrachy applicators was positioned on the same point. Then the orientation of the applicators was aligned to the direction of the HDR-BT catheters. Note that in this case, the applicators are not all parallel to each other.

For the SAR calculations, only calculation method 4 was used, and the electric field resulting from each electrode was calculated separately, as described in Section 2.2.4. The polarities and amplitudes of the electrodes were manually adjusted to produce a well-distributed temperature in the prostate tissue and a low temperature in the organs at risk, following the ESHO guidelines for IHT [13]. Namely, the temperature was not allowed to exceed 47 °C in any tissue, and the maximum temperature in the urethra, bladder, and rectum were set to a maximum of 43.5 °C, 42.5 °C, and 41.5 °C, respectively. For the optimization process, calculation method 4 was used for fast feedback of the adjustments. The temperature distribution was calculated from the resulting HT SAR distribution by solving Pennes’ bioheat equation [32] using the material properties stated in Table 1.

## 3. Results

### 3.1. SAR Calculation Benchmarking Results

**Figure 4:**
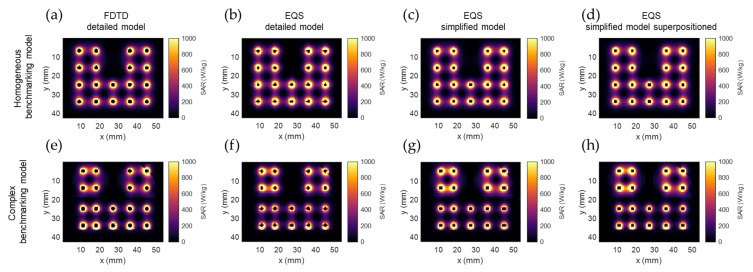
SAR calculation results of the different calculation methods in the two benchmarking models: (**a**,**e**) FDTD detailed model (calculation method 1); (**b**,**f**) EQS detailed model (calculation method 2); (**c**,**g**) EQS simplified model (calculation method 3); (**d**,**h**) superpositioned EQS simplified model (calculation method 4). The images show an axial slice passing through the center of the proximal electrode, as denoted with the green plane in Figure 3.

Figure 4 shows the SAR calculation results of the different calculation methods in the two benchmarking models. The four columns of the figure correspond to the four calculation methods from Section 2.2. It is evident that the SAR drops exponentially around each electrode. The SAR distribution around each electrode is also affected by differences in tissue properties and, as a secondary effect, by the distance between electrodes. In Figure 4e–h, it is visible that in the upper-right corner, the fat-prostate border (as seen in Figure 3D) leads to a discontinuity in SAR. The same holds for the horizontal border in the lower row of applicators (as seen in Figure 3E). About the effect of distance between electrodes, we see that the larger distance between row 2 and 3 in the y axis of Figure 4e–h compared to Figure 4a–d, leads to a drop in SAR, while the shorter distance between rows 1 and 2 in the x-axis leads to a higher SAR density. Between different calculation methods, it can be seen in Figure 4c,d,g,h that the applicators have a rectangular shape, rather than circular in Figure 4a,b,e,f. Other than that, no obvious differences can be noted.

Table 3 summarizes the memory and time requirements for all calculations. The computation domain is much larger for calculation method 1, as the whole applicator needs to be modeled to properly include the connection to the edge source. In the EQS calculation methods, the required computation domain is limited to the volume of interest stretched by a 10 mm margin, as explained in the materials and methods section. In terms of model voxel size, the simplified model in calculation methods 3 and 4 vastly reduces the necessary amount of voxels from more than 40 million and more than 30 million for detailed model calculation methods 1 and 2, respectively, to around 1 million voxels. This change is reflected by a significantly reduced model generation time from approximately 10 s to about 0.3 s. The simulation time needed for both benchmarking models in calculation method 1 was around 12 h, which is not feasible for treatment planning. The simulation time for calculation method 2 was already a lot shorter than that, with each simulation taking less than 10 min.

The SAR distribution resulting from superpositioning the electric field of multiple single-electrode calculations in calculation method 4 leads to identical simulation results as calculation method 3, thereby justifying the superpositioning approach. The time needed for a single electrode simulation was 13 ± 1 s (mean ± std) and 13 ± 0 s (mean ± std) for the homogenous and complex benchmarking model, respectively. This is similar to the simulation time of 13 s needed with calculation method 3.

### 3.2. Evaluation of SAR Calculations

**Figure 5:**
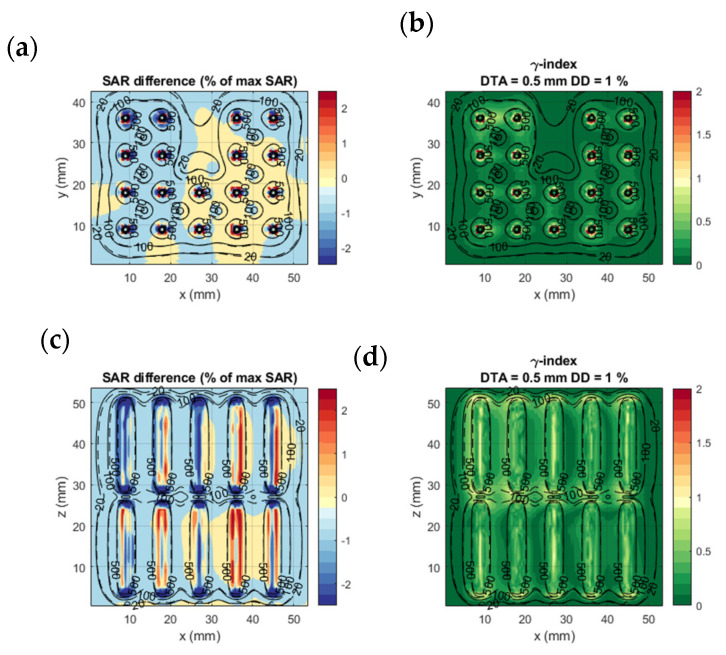
Homogenous benchmarking model: SAR difference between calculation method 1 and 4 (**a**,**c**) and γ-index of calculation method 4 with calculation method 1 results as a reference (**b**,**d**). (**a**,**b**) show the axial slice passing through the middle of the proximal electrode (green plane in Figure 3); (**c**,**d**) the coronal slice passing through the center of the lower row of electrodes. The dashed and solid black lines are isodose curves of the SAR calculated with calculation models 1 and 4, respectively.

Figure 5 shows the comparison of calculation method 1 and calculation method 4 in an axial (xy) and a coronal (xz) slice on the homogenous benchmark model. In Figure 5a,c, we notice that the most significant SAR value differences occur in the regions very close to the applicator. Other than that, the SAR differences between the two methods remain very low, with an accuracy (mean absolute error) of 0.50% of the maximum SAR and a bias (mean error) of −0.08% of the maximum SAR. The γ-index visualized in Figure 5b,d is higher in the regions of the applicators between the two electrodes. Moreover, calculation method 4 agrees with the FDTD detailed model calculations, with a γ-index 1%/0.5 mm DD and DTA passing rate of 99.2%. This result is remarkable, given the rigorous γ-index criteria.

**Figure 6:**
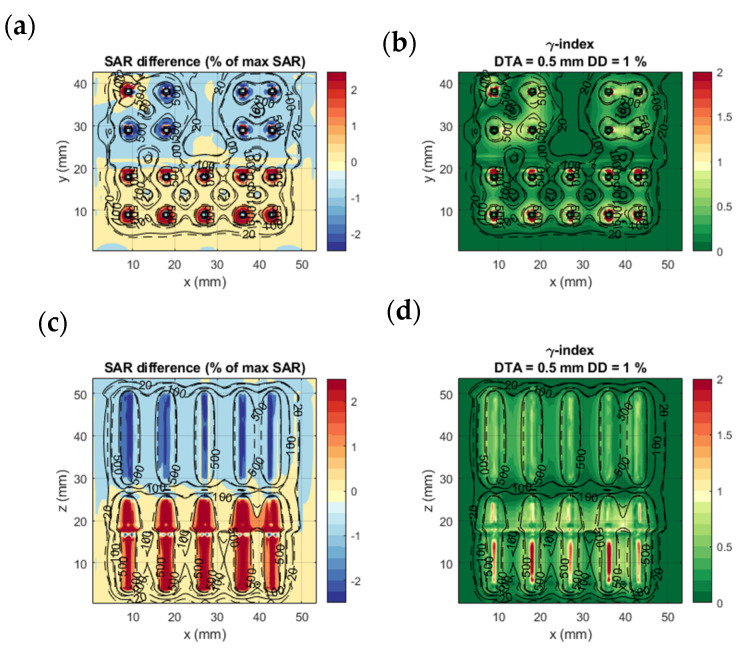
Complex benchmarking model: SAR difference between calculation method 1 and 4 (**a**,**c**) and γ-index of calculation method 4 with calculation method 1 results as a reference (**b**,**d**). (**a**,**b**) show the axial slice passing through the middle of the proximal electrode (green plane in Figure 3); (**c**,**d**) are coronal slices passing through the center of the lower row of electrodes. The dashed and solid black lines are isodose curves of the SAR calculated with calculation methods 1 and 4, respectively.

Figure 6 shows the comparison of calculation method 1 and calculation method 4 on the complex benchmark model. Likewise Figure 5, the SAR difference remains in the region within 1 mm from the applicators and is in very good agreement elsewhere. Although the tissue and geometric inhomogeneities affect the SAR distribution, the two calculation methods are in good agreement with each other. The accuracy remains good at 0.34% of maximum SAR and bias at 0.08% of maximum SAR. The γ-index showed a 1%/0.5 mm DD and DTA passing rate of 99.2%, which is as high as the results for the homogeneous model.

Table 4 shows the evaluation results for all calculation methods on the two benchmarking models. Similar to calculation methods 3 and 4, calculation method 2 shows good agreement with calculation method 1 in terms of accuracy, bias, and γ-index scoring. The results of calculation method 4 coincide with calculation method 3. This confirms that we can calculate the electric fields for each electrode separately and later combine them to generate the SAR distribution.

### 3.3. Treatment Planning Results in Patient Models

**Figure 7:**
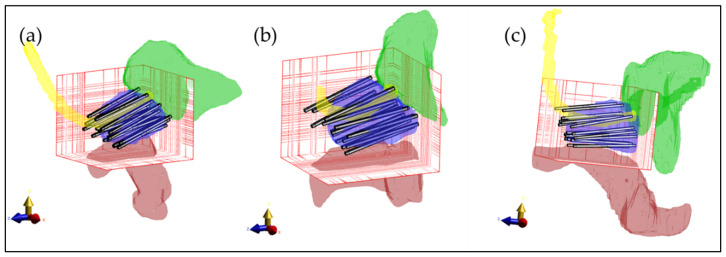
Three-dimensional visualization of the patient models with the applicators inserted in the prostate. (**a**) First patient with 18 implanted applicators; (**b**) Second patient with 17 implanted applicators; (**c**) Third patient with 19 implanted applicators. For visualization purposes, only the prostate (blue), rectum (red), urethra (yellow), and bladder (green) are visible. The bars represent the electrodes inserted in the prostate. The red grid represents the simulation domain.

**Figure 8:**
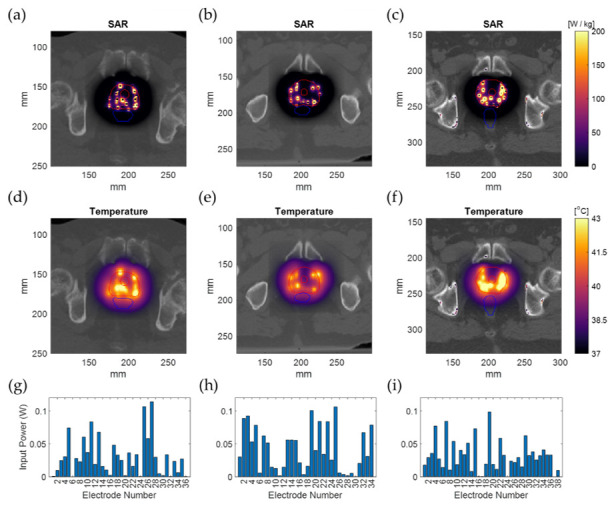
Simulation results for the real patient examples overlaid on the patient CT image. The SAR distribution (**a**–**c**); The temperature distribution after 20 min of heating (**d**–**f**); The input power applied on each implanted electrode (**g**–**i**). The outer and inner red lines represent the prostate and urethra contour, and the blue lines represent the rectum contour.

Calculation method 4 was used to calculate and optimize the HT plan in 3 patient models. The tissue model was generated from the CT imaging information, and the applicator positions were defined as in Figure 7. The electric field distribution was calculated for each separate electrode. The number of voxels of the resulting patient models was between 15.5 and 22.0 million. For each simulation (each electrode), model generation took 11.7 ± 0.2 s for the smaller model (Figure 7a) and 19.4 ± 0.2 s for the larger model (Figure 7c). The simulation time for these patient implantations was 152 ± 14 s per simulation for the smaller model and 230 ± 56 s per simulation for the larger model. The polarity and amplitude of each electrode were manually adjusted to get a well-distributed SAR distribution inside the prostate, as is shown in Figure 8a–c. The temperature distribution after 20 min of heating with the SAR distribution is visualized in Figure 8d,e. The applied power levels are presented in Figure 8f–i. For all patient models, the applied power levels ranged between 0 and 0.11 W, and the applied potential at the electrodes ranged between 0 and 5.2 V.

## 4. Discussion

Treatment planning in IHT is rarely used in clinical practice. It is regarded as complex due to the small and irregularly implanted structures of the HT applicators [13]. This becomes even more problematic, considering that calculations can only start once the applicators or catheters have been implanted into the patient since beforehand, the applicator positions are not known. One option to bypass those difficulties is to define the applicators in a grid-independent format, as was done by de Bree et al. [15,33]. However, this option is not always available in commercial simulation platforms. Another option is to apply the simulations on a model with an unstructured tetrahedron mesh to bypass the geometric issues. One drawback of this approach is that it is more computationally intensive to model complex anatomy in tetrahedrons, especially given that the anatomy is by definition imaged and generated in voxel format (CT, MRI, etc.), as is the computed radiation dose. The latter also raises compatibility issues with other anatomy and implant data, requiring transformations between a voxel-based and a tetrahedron-based space. Therefore, a more practical approach is to simplify the model of the applicators to an equivalent model in a computationally efficient way. In our study, we performed a geometric simplification as well as an EM simplification (EQS approximation).

We showed that by using an appropriate calculation method and by carefully applying simplifications, we could efficiently predict the SAR distribution of the IHT applicators and use it for online patient-specific treatment optimization similar to HDR-BT treatment planning regarding speed and accuracy.

In this study, we computed the SAR distribution in two benchmark models with different simulation methods. Comparing methods 1 and 2, we see that by using the appropriate simulation method, we can profit from an approximately 35 times faster calculation that does sacrifice accuracy (γ-index passing rate >99% for 1%/0.5 mm DD and DTA). In method 3, we replaced the applicator model with an analytically equivalent approximation. By replacing the cylindrical shape of the applicators with a rectangular shape, we obtained a model that is more convenient to use in a voxel-based environment. Furthermore, by adjusting the thickness and dielectric constant of the isolating material, we reduced the number of voxels for each applicator. The results were again highly comparable to the golden standard calculation method 1 (γ-index passing rate >99% for 1%/0.5 mm DD and DTA) and approximately 2000 times faster. In calculation method 4, we calculated the electric field of each electrode separately and combined the electric fields to generate the resulting SAR distributions. The almost equivalent results for calculation methods 3 and 4 verify the linearity of the EM field and prove that we can optimize the electrode parameters without a need for continuous recalculation of the SAR distributions, i.e., using an optimization algorithm like in [34,35].

The 1%/0.5 mm DD and DTA >99% agreement of this study is considerably tighter than the agreement considered acceptable in HDR-BT, where 2%/2 mm is regarded as a good agreement [31]. In our case, the 2%/2 mm agreement is >99.5%, as can be seen in Table 4. This pinpoints the macroscopic accuracy of our simplifications. The highest mismatch between calculation methods was close to and at the applicator surface. For calculation methods 3 and 4, this should be expected due to the different local geometry of the electrode and dielectric. The higher mismatch regions are also the regions where the SAR is highest. As can be observed in Figure 8, the almost exponential SAR drop around the applicators in Figure 8a–c translates into a less steep and broader temperature distribution around the applicators in Figure 8d–f. This is also evident and experimentally verified in our earlier work about the ThermoBrachy applicators [12]. Hence, the impact of calculation inconsistencies can be presumed to be lower for the temperature distribution.

In the framework of simultaneous ThermoBrachytherapy, it is crucial to generate an IHT treatment plan in a time frame of minutes for the patient who has been implanted with applicators. Treatment planning calculation time in BT takes approximately up to 15 min [36]. In this study, we performed a single calculation of the electric field and SAR for three patient implantations in 2.5 to 4 min on a standard PC. This can be further reduced to seconds by using better hardware, parallelization, and application of specific software optimization. Nevertheless, the presented clinical example IHT planning results demonstrate that it is feasible to perform IHT treatment planning in a timeframe that fits well within the standard HDR workflow. The feasibility of electric field superpositioning also enables fast, automated SAR or temperature-based optimization of the IHT treatment, improving the IHT treatment planning quality even further. In adaptive treatment planning, artificial intelligence might play a role as well [37]. With calculation methods 3 and 4, the computational complexity of the EM field is scaled down substantially. The approach can be used for rapid computation of the EM field. This is, therefore, a significant improvement towards parallel IHT and HDR-BT planning.

## 5. Conclusions

This study demonstrates that it is feasible to perform fast and accurate treatment planning for the capacitively coupled ThermoBrachy applicators operating at 27 MHz using commercial treatment planning software. By using a quasistatic approximation and applying a simplified applicator geometry, the computation time of a realistic IHT applicator configuration can be reduced from hours to seconds without losing calculation accuracy. The proposed hyperthermia treatment planning approach has the potential to integrate into the standard HDR-BT workflow.

## Figures and Tables

**Table 1:** Electric and Thermal properties of the applicator materials and the tissues used in the simulations.

Tissue	Mass Density (kg/m^3^)	Electric Conductivity at 27 MHz (S/m)	Relative Permittivity at 27 MHz	Specific Heat Capacity (J/kg/K)	Thermal Conductivity k (W/m/K)	Perfusion Rate (ml/kg/min)
POM [21]	1150	2.7 × 10^−5^	3.6	1670	0.230	-
Parylene C [22,23]	1289	1 × 10^−5^	2.4	712	0.084	-
Air [24]	1.164	0	1	1004	0.0273	-
Muscle [24]	1090.4	0.654	95.764	3421	0.495	39.74
Fat [24]	911	0.061	17.928	2348	0.211	32.71
Prostate [24]	1045	0.838	120.056	3760	0.512	394.12
Rectum [24]	1045	0.654	95.8	3801	0.557	0
Urethra [24]	1102	0.375	88.8	3306	0.462	394
Bladder [24]	1086	0.276	31.5	3581	0.522	78

**Table 2:** Overview of calculation methods evaluated with the benchmarking models.

	Calculation Method 1	Calculation Method 2	Calculation Method 3	Calculation Method 4
Numerical computation method	FDTD	FEM	FEM	FEM
Physics model	Maxwell’s curl equations	Electroquasistatic approximation	Electroquasistatic approximation	Electroquasistatic approximation—E-fields Superpositioning
Geometric model	Detailed TBT structure	Detailed TBT structure	Rectangular simplified TBT model	Rectangular simplified TBT model
Evaluated quantity	SAR	SAR	SAR	SAR

**Table 3:** Table summarizing the memory and time requirements of all evaluated calculation methods on the homogenous and complex benchmarking model.

Calculation Method	Single FDTD Detailed Model (Calculation Method 1)		Single EQS Detailed Model (Calculation Method 2)		Single EQS Simplified Model (Calculation Method 3)		Superpositioned EQS Simplified Model (Calculation Method 4)	
Model	Homogeneous	Complex	Homogeneous	Complex	Homogeneous	Complex	Homogeneous	Complex
simulation domain (cm^3^)	2 880	2 880	345	345	345	345	345	345
number of voxels (10^6^)	41.789	42.322	31.370	32.173	1.14	1.12	36 × 1.14	36 × 1.12 ^†^
model generation time	10.06 s	10.10 s	7.61 s	8.79 s	0.30 s	0.29 s	36 × 0.30 s ^†^	36 × 29 s ^†^
simulation time	11 h 43 min *	11 h 45 min *	8 min 35 s **	9 min 37 s **	13 s **	13 s **	36 × 13 s ^†‡^**	36 × 13 s ^†‡^**

* Time using the CUDA GPU accelerated calculation algorithm, utilizing three GeForce GTX 1080 Ti graphics cards. ** Time using CPU calculations on an Intel Core i7-6700 CPU. ^†^ The voxels/time needed for one simulation multiplied by the number of separate electrodes. ^‡^ The actual time for each separate simulation varied slightly, but the average was equal to this value.

**Table 4:** Summary of evaluation results of the three EQS calculation methods compared to the golden standard FDTD calculation method on the homogeneous and complex benchmarking models.

Calculation Method	Single EQS Detailed Model (Calculation Method 2)		Single EQS Simplified Model (Calculation Method 3)		Superpositioned EQS Simplified Model (Calculation Method 4)	
Model	Homogeneous	Complex	Homogeneous	Complex	Homogeneous	Complex
Accuracy (% of max SAR)	0.52	0.30	0.50	0.34	0.50	0.34
Bias (% of max SAR)	−0.01	0.13	0.22	0.08	0.22	0.08
2%/2 mm γ-index passing rate (%)	99.8	99.8	99.6	99.7	99.6	99.7
1%/0.5 mm γ-index passing rate (%)	99.6	99.3	99.2	99.2	99.2	99.2

## Data Availability

The data presented in this study can be made available upon request to the corresponding author.
